# From Pain to Clarity: A Case of Pneumoperitoneum Diagnosed by Point-of-care Ultrasound

**DOI:** 10.5811/cpcem.50858

**Published:** 2026-08-11

**Authors:** Archana Nair, Soubhik Chakraborty, Tej Prakash Sinha, Sanjeev Bhoi

**Affiliations:** All India Institute of Medical Sciences, Department of Emergency Medicine, New Delhi, India

**Keywords:** point-of-care ultrasound, intestinal perforation, pneumoperitoneum, case report

## Abstract

**Introduction:**

Acute abdomen is a common emergency presentation, and gastrointestinal perforation can be rapidly fatal if not promptly identified. Point-of-care ultrasound (POCUS) offers a rapid bedside alternative when advanced imaging is delayed or unavailable.

**Case Report:**

We report a 40-year-old man presenting in cardiac arrest, resuscitated after ten minutes, with subsequent findings of abdominal distension and prior epigastric pain. Point-of-care ultrasound demonstrated classic signs of pneumoperitoneum, and intestinal perforation was confirmed by the drainage of feculent fluid. Despite intervention, the patient expired.

**Conclusion:**

This case underscores the value of POCUS in diagnosing perforation in unstable patients, guiding emergent decisions when transfer or definitive imaging is not feasible.

## INTRODUCTION

Undifferentiated abdominal pain is a commonly encountered emergency. It requires prompt differentiation between medical and surgical causes. While initial evaluation is guided by history and physical examination, establishing a definitive diagnosis in patients with abdominal pain generally requires adjunctive laboratory and imaging studies. Increasingly, point-of-care ultrasound (POCUS) is being recognized as an effective, rapid diagnostic modality capable of identifying life-threatening intra-abdominal conditions, reducing reliance on radiologic imaging, and expediting clinical decision-making. The sonographic evaluation for pneumoperitoneum using POCUS is relatively straightforward but highly dependent on the operator’s proficiency. This case review illustrates the clinical utility of abdominal POCUS in diagnosing pneumoperitoneum and supports its broader integration into the evaluation of acute abdominal presentations. The integration of multiple classic sonographic signs-including enhanced peritoneal stripe sign, abdominal A-lines, absent sliding, and the gut point, enabled rapid bedside diagnosis and directly guided emergent intervention. This case reinforces the value of POCUS not only as a diagnostic modality but also as a critical decision-making tool in time-sensitive, resource-limited scenarios. Furthermore, it highlights the importance of considering intra-abdominal causes in non-traumatic cardiac arrest, an area that may be under-recognized in emergency practice.

## CASE REPORT

A 40-year-old male with no prior comorbidities presented to the emergency department in cardiac arrest. Following advanced cardiac life support, return of spontaneous circulation was achieved after approximately 10 minutes. Post-resuscitation care was initiated. Detailed clinical history revealed epigastric pain two days prior. On examination, his abdomen was distended and rigid.

Point-of-care ultrasound was performed and demonstrated the following key findings: An enhanced peritoneal stripe sign (Image 1), absent gut sliding (gut barcode sign) (Image 2), abdominal Aprofile (Image 3) and free fluid containing echogenic particulate matter (Image 4), all consistent with intestinal perforation with pneumoperitoneum.

Subsequent formal abdominal ultrasound confirmed pneumoperitoneum, most notably via the enhanced peritoneal stripe sign—a hyper-echoic thickening of the peritoneal stripe indicative of free intraperitoneal air.^1^

Due to hemodynamic instability requiring dual vasopressor support, the patient was deemed unfit for transfer to the operating theatre for exploratory laparotomy. Ultrasound findings prompted immediate bedside intervention with a 22 French drain, yielding approximately 2500 mL feculent fluid—a temporizing measure given the inability to reach operation theatre. Abdominal radiography corroborated the ultrasound findings, demonstrating subdiaphragmatic free air and dilated bowel loops (Image 5).

*CPC-EM Capsule*

What do we already know about this clinical entity?

*Pneumoperitoneum from gastrointestinal perforation is life-threatening.*

What makes this presentation of disease reportable?

*Point-of-care ultrasound (POCUS) enabled a rapid diagnosis in an unstable patient, enabling bedside drainage when operating room transfer was impossible.*

What is the major learning point?

*Integrate multiple POCUS signs—E-point septal separation, A-lines, gut point, and absent sliding—to diagnose pneumoperitoneum in unstable patients before imaging.*

How might this improve emergency medicine practice?

*Routine POCUS for acute abdomen can expedite diagnosis of perforation, guide bedside intervention, and reduce delays when transfer is not feasible.*

The patient was started on broad spectrum antibiotic coverage and other supportive measures. Despite aggressive resuscitation patient expired. This case emphasizes the importance of point-of-care ultrasound in diagnosing a perforation peritonitis and pneumoperitoneum.

## DISCUSSION

Abdominal ultrasound is performed with a low frequency abdominal probe. It is positioned horizontally with pointer towards right side of the patient. Numerous parallel, equidistant, horizontal hyperechoic artifacts can be seen extending down the image, with an absence of identifiable abdominal structures. These are similar to the reverberation artefacts of pleura in lung ultrasound, which are called as A lines. Therefore, these reverberation artefacts due to presence of pneumoperitoneum are called abdominal A lines.^2^

A highly echogenic peritoneal stripe is evident at the junction between the parietal peritoneum and intraperitoneal free air. This appearance is due to the marked acoustic impedance mismatch between soft tissue and free intraperitoneal air, which causes near-total reflection of ultrasound waves at the peritoneal interface, resulting in a brightly echogenic stripe and associated reverberation artifacts. Multiple repeating horizontal artefacts, corresponding to reverberation of the peritoneal line due to free intra-abdominal air, are present. The ‘gut point’ refers to the transition zone where the sliding motion of the parietal peritoneum ceases and these reverberation artefacts begin to appear. These ultrasound findings closely resemble those seen in thoracic air leaks, such as the presence of A-lines and the absence of pleural sliding on lung ultrasound.^3^ Abdominal free air produces a strong linear echo with a pathognomonic peritoneal stripe sign.

Gut point is analoguous to lung point of pneumothorax.^4^ A diagnostic hallmark is the identification of the “gut point,” which represents the interface between dynamic abdominal wall sliding and a static, non-sliding area, signifying the presence of free air. Normally, a subtle shimmering or sliding movement along the peritoneal line signifies the normal apposition of the visceral and parietal peritoneum. The abnormal accumulation of free intraperitoneal air disrupts this interface, resulting in the separation of these peritoneal layers.

Despite its utility, POCUS for pneumoperitoneum is subject to potential pitfalls. Intraluminal bowel gas can mimic free intraperitoneal air by producing echogenic interfaces and reverberation artifacts, potentially leading to false-positive interpretations. However, in such cases, the echogenic line typically corresponds to the bowel wall and is associated with an overlying hypoechoic bowel wall layer and visible peristalsis. In contrast, free intraperitoneal air accumulates immediately beneath the anterior abdominal wall, producing an enhanced peritoneal stripe without an intervening hypoechoic layer and often demonstrating positional changes with patient movement. Careful attention to probe positioning, dynamic assessment, and identification of the “gut point” can further improve diagnostic accuracy and reduce misinterpretation.

## CONCLUSION

This case illustrates the power of pointofcare ultrasound in early detection of perforation peritonitis and pneumoperitoneum. Computed tomography remains gold standard, but POCUS offers rapid, nontransfer-dependent bedside diagnosis—critical in unstable patients. Given its excellent sensitivity and specificity, affordability, and favorable safety profile, abdominal ultrasound ought to be regarded as the initial imaging choice for detecting pneumoperitoneum and should be broadly adopted by emergency medicine physicians.

## Figures and Tables

**Image 1:**
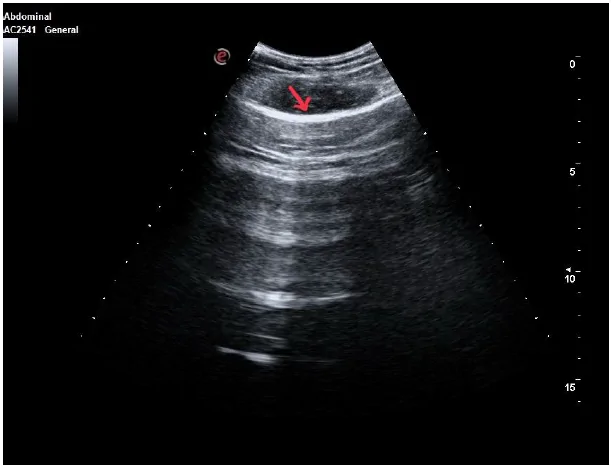
Enhanced peritoneal stripe sign (arrow) in a patient with acute abdominal pain indicative of free peritoneal air.

**Image 2:**
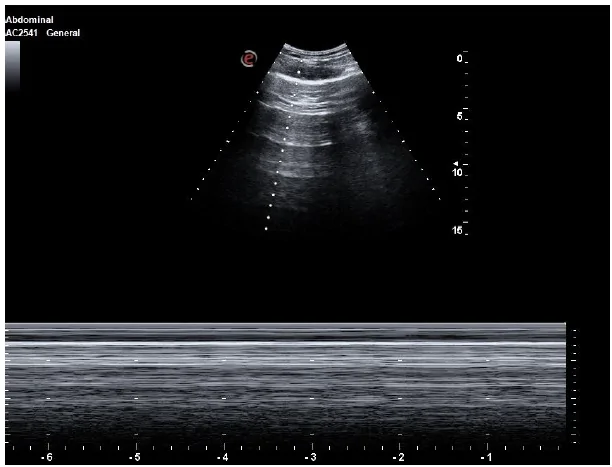
Absent gut sliding (gut barcode sign) in M mode in a patient with acute abdominal pain signifying the presence of free air.

**Image 3:**
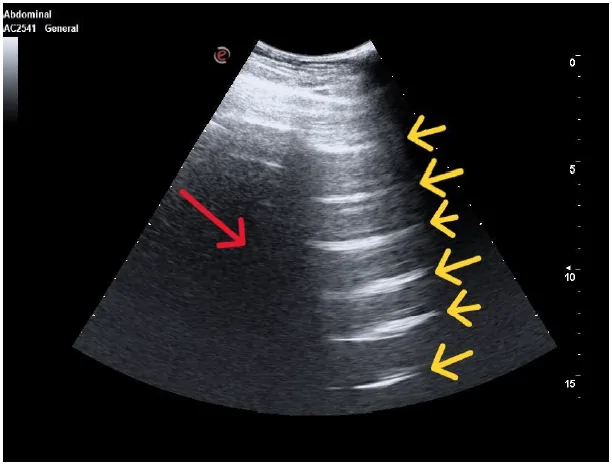
Abdominal A profile with right half not showing A lines because of the liver. Red arrow shows liver and yellow arrows shows A lines, in a patient with acute abdominal pain suggesting pneumoperitoneum.

**Image 4:**
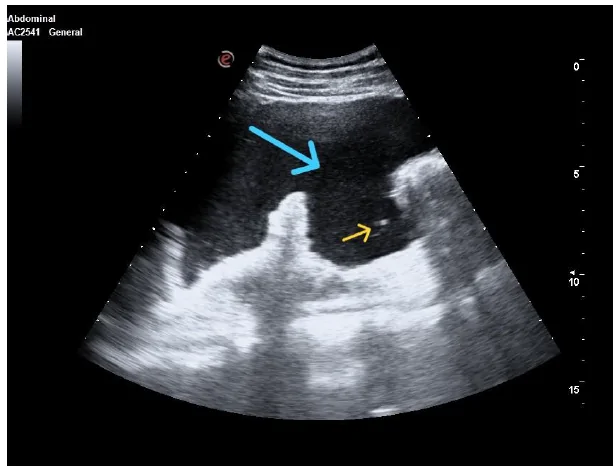
Free fluid containing echogenic particulate matter. Blue arrow shows the free fluid and yellow arrow shows particulate matter, in a patient with acute abdominal pain, indicative of intestinal perforation.

**Image 5:**
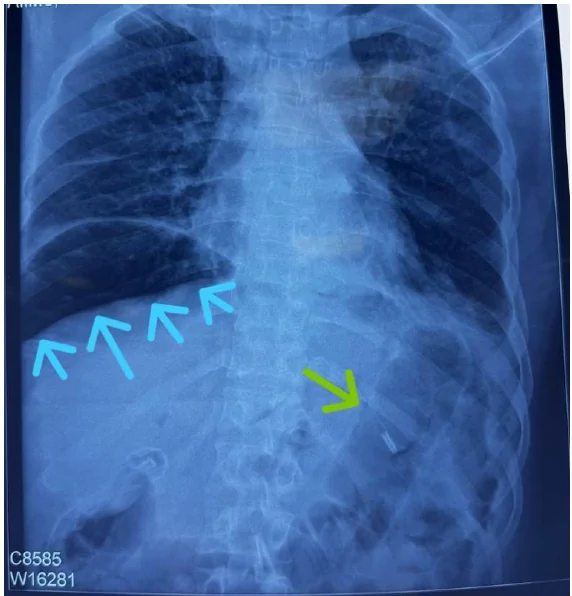
Abdominal radiograph demonstrating subdiaphragmatic free air and dilated bowel loops in a patient with acute abdominal pain. Blue arrows show air under diaphragm and green arrow shows dilated bowel.
